# Optimal Range of Lymphadenectomy in Pathological Stage T1 and T2 Esophageal Squamous Cell Carcinoma

**DOI:** 10.3389/fonc.2021.619556

**Published:** 2021-05-25

**Authors:** Hansheng Wu, Weitao Zhuang, Shujie Huang, Xueting Guan, Yuju Zheng, Zefeng Xie, Gang Chen, Jiming Tang, Haiyu Zhou, Liang Xie, Xiaosong Ben, Zihao Zhou, Zijun Li, Rixin Chen, Guibin Qiao

**Affiliations:** ^1^ Department of Thoracic Surgery, The First Affiliated Hospital of Shantou University Medical College, Shantou, China; ^2^ Department of Thoracic Surgery, Guangdong Provincial People’s Hospital, Guangdong Academy of Medical Sciences, Guangzhou, China; ^3^ Shantou University Medical College, Shantou, China; ^4^ Research Center of Medical Sciences, Guangdong Provincial People’s Hospital, Guangdong Academy of Medical Sciences, Guangzhou, China

**Keywords:** lymphadenectomy, surgical strategy, prognosis, negative lymph node, esophageal squamous cell carcinoma (ESCC)

## Abstract

**Background:**

Lymph node metastasis is a primary contributor to tumor progression in esophageal squamous cell carcinoma (ESCC), and the optimal extent of lymphadenectomy during esophagectomy remains controversial. This study aimed to investigate the appropriate number of lymph nodes to be dissected in pT1-2Nany stage ESCC to achieve the best prognosis and avoid missing positive lymph nodes (PLNs).

**Methods:**

A total of 497 patients with pT1 to pT2 esophageal cancer from two institutions were retrospectively analyzed and their surgical and pathological records were critically reviewed. Stepwise analyses were conducted by calculating a serial of hazard ratios and odd ratios to determine the optimal range of lymphadenectomy for overall survival (OS).

**Results:**

The best survival outcome can be obtained when the number of lymph node examined (NLNE) is 10–18 in pT1N0 ESCC, while the NLNE should exceed 24 in pT2N0 diseases. In patients with pT1-2Nany and pT2Nany ESCC, resection of 15–25 and 24–37 lymph nodes, respectively, could provide significant added value for identifying positive nodal metastasis. When the NLNE exceeds this appropriate range, resection of extra lymph node is not helpful to improve the probability of finding PLNs.

**Conclusions:**

For ESCC patients undergoing radical esophagectomy, the optimal extent of lymphadenectomy is 15–25 for pT1Nany disease and 24–37 for pT2Nany disease.

## Introduction

Esophageal cancer (ESCA) is a highly invasive and lethal disease that accounts for more than 400,000 deaths/year worldwide (1, 2), with the 5-year overall survival (OS) rate being less than 30% (3). The status of lymph node metastasis, one of the cornerstones for pathological staging, is the key prognostic factor for survival outcome of ESCA (4, 5). A two-field or three-field lymphadenectomy has been widely accepted during esophagectomy. However, the optimal extent of lymphadenectomy in patients with relatively early-stage ESCA that have a low probability of node metastasis is still controversial (6, 7).

Several previous studies have confirmed the association between the number of resected lymph nodes and the prognosis of ESCA (8–10). According to the current clinical guidelines, extensive lymphadenectomy can provide survival benefits and is hence considered the gold standard of treatment (11). It was suggested that the number of negative lymph nodes (NLNs) was an independent prognostic factor for patients with thoracic esophageal squamous cell carcinoma (ESCC) after radical esophagectomy (12), wherein a higher ratio of NLNs was independently associated with better OS (13). Except for the number, resection of certain lymph node stations might also matter in survival outcome based on the analysis of index of estimated benefit from lymph node dissection (IEBLD) (14, 15). Nevertheless, extensive lymphadenectomy is usually accompanied with additional surgical complications such as paralysis of the recurrent laryngeal nerve, intraoperative hemorrhage, and pulmonary complications that may lead to unfavorable short-term outcomes and largely impair the quality of life of patients (16, 17). Interestingly, a large-scale study on 1044 patients from Sweden has shown that more extensive lymphadenectomy could not reduce mortality in ESCA of any specific T stage (18). Therefore, it is necessary to further explore the optimal extent of lymph node resection, not only to achieve a better survival outcome but also to avoid possible missing of positive lymph nodes. For patients with stage T3 or T4 tumors, which are associated with a much higher chance of lymph node metastasis than other stages, systemic lymphadenectomy should be performed when possible. However, the optimal range of lymph node resection in T1 or T2 ESCC remains controversial, and to our knowledge, no consensus has yet been reached (12, 13, 18–20).

In this study, we retrospectively analyzed ESCC patients with pathological stage T1-2NanyM0 from two thoracic surgical institutions. We aimed to determine the optimal extent of lymphadenectomy and provide more evidence as a reference for thoracic surgeons when performing esophagectomy.

## Patients and Methods

### Patients

**Figure 1:**
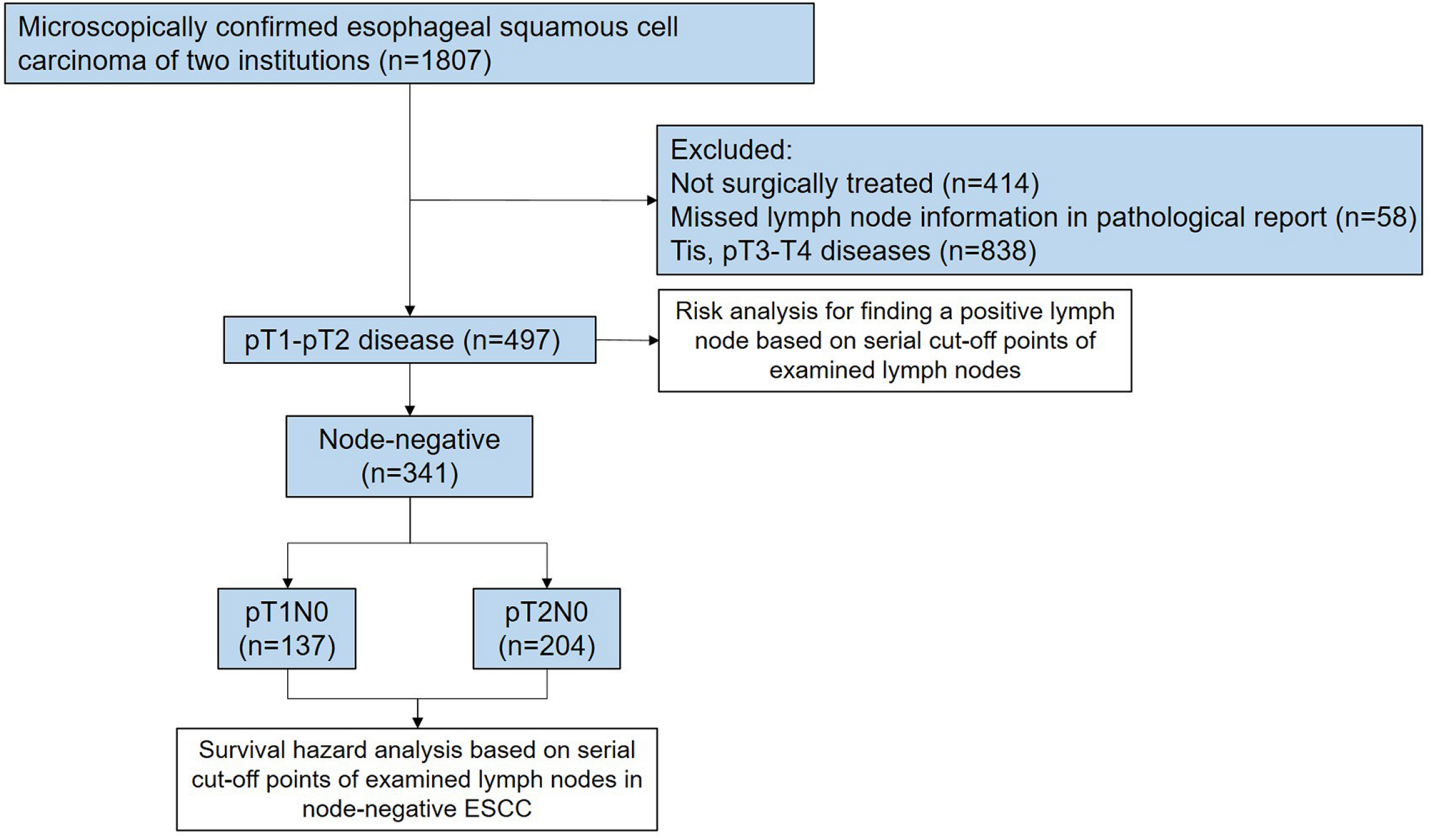
Diagram of patient selection and analysis.

Two ESCC databases consisting of 1,807 patients were accessed between 2009 and 2019 in Guangdong Provincial People’s Hospital and The First Affiliated Hospital of Shantou University Medical College to identify the study cohort. These two databases have been prospectively maintained by a regular extraction and review of general information and clinical data from medical records. The eligibility criteria of patients in this retrospective analysis included: (i) pathologically confirmed diagnosis of ESCC; (ii) pathological stage of ESCC ranging from T1NanyM0 to T2NanyM0; (iii) surgically treated with a curative intent; (iv) no history of neoadjuvant therapies if postoperative node-negativity confirmed; and (v) patient age from 18 to 80 years. Patients with suspicious lymph nodes preoperatively were also included. Completeness of the surgical records and pathological reports was assessed to exclude any patients with missed lymph node information. Node-negative patients who received neoadjuvant therapies were excluded to eliminate the effect of lymph node downstaging, which ensured the “true negative” status of these patients. A total of 497 patients were finally included for this retrospective analysis; of these, 341 cases were node-negative ESCC (**Figure 1**). This study was approved by the Institutional Review Boards of two medical centers (No. GDREC2019687H and No. 2020-094).

### Preoperative Examinations

The institutional protocol of preoperative workup for ESCC patients in both medical centers included barium swallow, esophageal endoscopy with biopsy, contrast-enhanced computed tomography (CT) of the chest and abdomen, transthoracic echocardiography, and pulmonary function test. Since 2015, whole body PET/CT was recommended for each patient if they could afford the costs.

### Surgical Procedures, Lymphadenectomy, and Pathological Examination

The two involved institutions, Guangdong Provincial People’s Hospital and The First Affiliated Hospital of Shantou University Medical College, implement a uniform surgical strategy, with McKeown/minimally invasive McKeown esophagectomy being the most common procedure for the upper and middle thoracic tumors. Ivor–Lewis/minimally invasive Ivor–Lewis or Sweet esophagectomy might also be performed depending on the tumor’s location or clinical stage. Overall, at least a two-field lymphadenectomy was performed in all included patients. The resected lymph nodes included but were not limited to the para-trachea nodes (group 2R/2L/4R/4L), para-esophageal nodes (group 8U/8M/8Lo), subcarinal nodes (group 7), sub-aortic nodes (group 5), abdominal nodes (group 16/17), and supraclavicular nodes (group 1L/1R). Lymph nodes that had been resected into several fragments were usually bagged together and counted as one single node during pathological examination. Pathological staging was carried out according to the 8th edition of the AJCC/UICC TNM classification system (21). The total number of lymph nodes was resected from the neck, chest, and abdomen. The pathological stage N0, N1, N2, and N3 were defined as 0, 1–2, 3–6, and ≥7 positive lymph nodes, respectively.

### Patient Follow-Up

The patients were scheduled for follow-up every 3 months in the first two years after esophagectomy and every 6 months in the following three years. Laboratory workup and radiological examinations included serum tumor biomarkers, nutritional indices, hepatic and renal function tests, and thoracic and abdominal CT scans. For convenience, some patients would complete their follow-up examinations in their local medical institutions. This group of patients were regularly contacted *via* telephone to record their health and vital status. The patients in our study group were last contacted on May 31, 2020. The median follow-up time of this study was 51.0 months (range: 2–130 months). The primary outcome of the current study was OS, which was defined as the duration from the date of esophagectomy to the date of death due to any cause. Patients who were lost to follow-up or were still alive after the cut-off date for follow-up were classified as censored data in the statistical analysis.

### Statistical Analysis

The patients were categorized according to their pathological T classifications and lymph node metastatic status. Categorical data were presented as frequency and percentage. Univariate analysis was performed by using log-rank test, and variables with a p value<0.15 were included in the multivariate analysis by using Cox proportional hazards regression. The distribution of positive and negative lymph node metastasis (LNM) in association with the number of lymph nodes examined (NLNE) was depicted by histograms. To determine the trend of survival outcome affected by the NLNE, a series of hazard ratios and their corresponding confidence interval were computed for multiple cut-off points using the Cox proportional hazards regression model. The method of locally weighted scatterplot smoothing (LOWESS) was used to fit the curve, and several ranges of NLNE were subsequently determined to distinguish patients with potentially different survival outcomes. Kaplan–Meier method and log-rank test were then applied for survival analysis. Pearson’s correlation analysis was performed between the number of resected lymph nodes and the number of resected stations, and Kruskal–Wallis test was used for comparison of number of resected stations among different ranges of resected lymph nodes. The odd ratios (ORs) of positive finding per lymph node examined for a series of cut-off points were calculated using crosstable and compared using Pearson’s chi square test, with the objective to determine the best effective range of lymphadenectomy.

## Results

### Patient Characteristics

**Figure 2:**
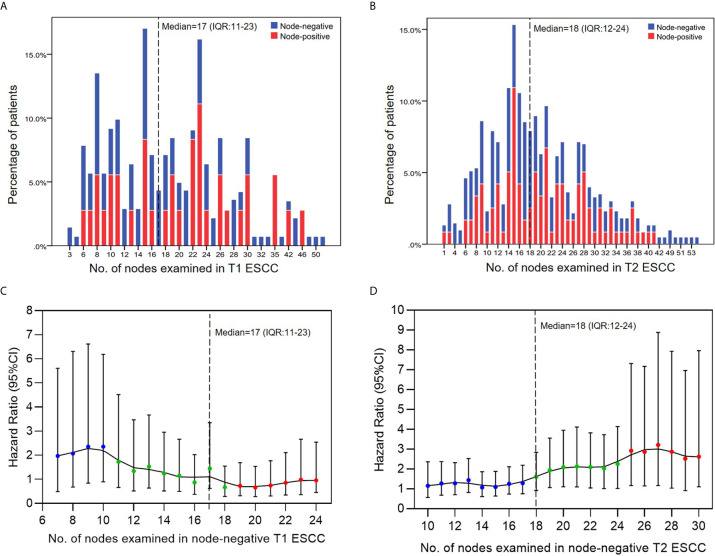
**(A, B)** The distribution of positive and negative findings associated with the number of nodes examined. **(C, D)** Survival hazard analysis based on multiple cut-off points of examined lymph nodes in node-negative ESCC. Hazard ratio represents the survival hazards on the left-side panel of cut-off point compared to the right-side panel of cut-off point.

According to the depth of invasiveness, the included ESCC patients were divided into two cohorts: pT1 cohort (n=174) and pT2 cohort (n=323). As summarized in **Table 1**, 78.7% (137/174) and 63.2% (204/323) patients were node-negative in the pT1 and pT2 cohorts, respectively. The majority of patients had ESCC on the middle thoracic esophagus (75.9% pT1 patients and 65.9% pT2 patients). According to the 8^th^ edition of TNM staging system, all pGxT1N0M0 (n=137) and pG1T2N0M0 (n=30) patients were staged as IB ESCC, whereas the pG2-3T2N0M0 (n=174) diseases were staged as IIA. Approximately three quarters of patients received the McKeown esophagectomy. More than 70% patients received minimally invasive esophagectomy (MIE). All node-negative patients included in our study did not receive neoadjuvant or adjuvant therapies. The distribution of positive and negative findings in lymph node examination are presented in **Figures 2A, B**. The median number of lymph nodes examined in pT1 and pT2 patients were 17 [interquartile range (IQR): 11–23] and 18 (IQR: 12–24), respectively.

**Table 1:** Clinicopathologic characteristics of pT1 and pT2 ESCC patients.

	Overall pT1 cohort (n=174) (%)	Overall pT2 cohort (n=323) (%)	pT1N0 (n=137) (%)	pT2N0 (n=204) (%)
**Age, years**				
≤60	88 (50.6)	148 (45.8)	64 (46.7)	98 (48.0)
>60	86 (49.4)	175 (54.2)	73 (56.3)	106 (52.0)
**Sex**				
Male	121 (69.5)	252 (78.0)	94 (68.6)	159 (77.9)
Female	53 (30.5)	71 (22.0)	43 (31.4)	45 (22.1)
**BMI (kg/m^2^)**				
<18.5	25 (14.4)	39 (12.1)	9 (6.6)	16 (7.8)
18.5–23.9	110 (63.2)	210 (65.0)	100 (73.0)	146 (71.6)
>23.9	39 (22.4)	74 (22.9)	28 (20.4)	42 (12.3)
**Charlson Comorbidity Index**				
0–1	151 (86.8)	293 (90.7)	122 (89.6)	188 (92.2)
≥2	23 (13.2)	30 (9.3)	15 (10.4)	16 (7.8)
**Tumor Location**				
Upper thorax	16 (9.2)	37 (11.5)	15 (10.9)	26 (12.7)
Middle thorax	132 (75.9)	213 (65.9)	102 (74.5)	132 (64.7)
Lower thorax	26 (14.9)	73 (22.6)	20 (14.6)	46 (22.6)
**Pathological Stage**				
IB	137 (78.7)	30 (9.3)	137 (100)	30 (14.7)
IIA	0	174 (53.9)	0	174 (85.3)
IIB	26 (14.9)	0	0	0
IIIA	8 (4.7)	61 (18.9)	0	0
IIIB	0	42 (13.0)	0	0
IVA	3 (1.7)	16 (4.9)	0	0
**Pathological N stage**				
N0	137 (78.7)	204 (63.2)	137 (100)	204 (100)
N1	26 (14.9)	61 (18.9)	0	0
N2	8 (4.7)	42 (13.0)	0	0
N3	3 (1.7)	16 (4.9)	0	0
**Tumor Grade**				
Well-differentiated	13 (7.5)	40 (12.4)	10 (7.3)	30 (14.7)
Moderately differentiated	132 (75.9)	219 (67.8)	102 (74.5)	143 (70.1)
Poorly or not differentiated	29 (16.7)	64 (19.8)	25 (18.2)	31 (15.2)
**Lymph node examined (T1/T2)**				
≤10/≤17	41 (23.6)	159 (49.2)	34 (24.8)	106 (52.0%)
11–18/18–24	57 (32.7)	78 (24.2)	48 (35.0)	48 (23.5)
>18/>24	76 (43.7)	86 (26.6)	55 (40.1)	50 (24.5)
**Surgical procedure**				
Sweet	16 (9.2)	34 (10.5)	16 (11.6)	20 (9.8)
Ivor–Lewis	26 (14.9)	37 (14.7)	23 (16.8)	23 (11.3)
McKeown	132 (75.9)	252 (74.8)	98 (71.6)	161 (78.9)
**Surgical approach**				
Open	38 (21.8)	82 (25.4)	31 (22.6)	44 (21.6)
Minimally invasive	136 (78.2)	241 (74.6)	106 (77.4)	160 (78.4)

### Range-Partition of NLNE

A series of consecutive cut-off points of NLNE were applied in node-negative ESCC for the calculation of survival hazards of a lower range of LNE compared to the higher range. For practicality, two cut-off values were determined by break points of the LOWESS curve to separate the NLNE into three different ranges (colored in blue, green, and red, respectively); these points were 10 and 18 in pT1 diseases and 17 and 24 in pT2 diseases (**Figures 2C, D**).

### Relationship Between the Number of Resected Lymph Nodes and Number of Stations

**Figure 3:**
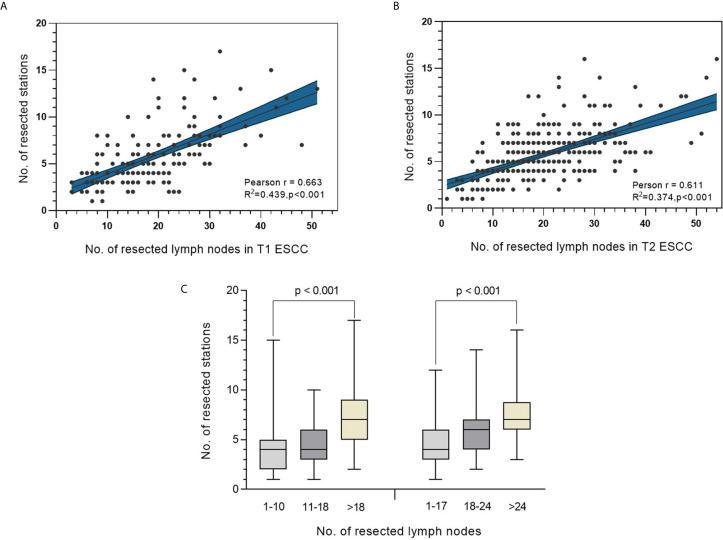
**(A, B)** Correlation analysis between number of resected lymph nodes and the corresponding lymph node stations in T1 and T2 ESCC. **(C)** Distribution of number of resected stations in different categories of resected lymph nodes in patients with T1 ESCC (left panel) or T2 ESCC (right panel).

A significant positive correlation between the number of resected lymph nodes and the number of lymph node stations was observed in both T1 and T2 ESCC patients, with Pearson’s correlation coefficients being 0.663 and 0.611, respectively (both p<0.001) (**Figures 3A, B**). Further comparison between categories based on the abovementioned ranges revealed a significantly greater number of resected stations as the number of resected lymph nodes increased (p<0.001 in both T1 and T2 ESCC patients) (**Figure 3C**).

### Prognostic Value and Optimal Range of NLNE

**Figure 4:**
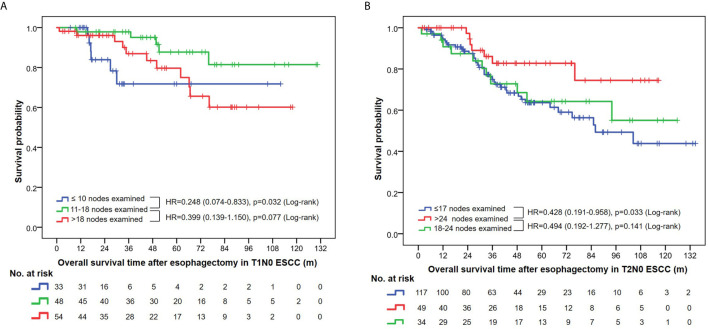
**(A)** Overall survival curves in T1N0 ESCC patients stratified by the number of nodes examined. **(B)** Overall survival curves in T2N0 ESCC patients stratified by the number of nodes examined.

Multivariate analysis using Cox proportional hazards regression demonstrated the prognostic value of the NLNE (**Table 2**). Although statistical significance was not reached in the overall analysis (p=0.074 and p=0.077 in T1N0 and T2N0 patients, respectively), an optimal range of LNE did exist in terms of a favorable OS in pT1N0 (NLNE 11–18, HR=0.249, 95%CI=0.074–0.838) and pT2N0 (NLNE>24, HR=0.449, 95%CI=0.196–0.921). Further survival analysis based on the abovementioned range of LNE revealed that a significantly better OS was obtained when dissecting 11–18 nodes in pT1N0 ESCC than when dissecting <11 nodes (HR=0.248, 95%CI=0.074–0.833, p=0.032) or >18 nodes (HR=0.399, 95%CI=0.139–1.150, p=0.077). Similarly, resection of >24 nodes could achieve a significantly better OS in pT2N0 diseases than that of <18 nodes (HR=0.428, 95%CI=0.191–0.958, p=0.033) (**Figure 4**). It is notable that patients with pT1N0 ESCC who had only ≤10 nodes removed had a sharp decline in the survival curve in the first 3 years after esophagectomy (**Figure 4A**). This suggested that an insufficient lymph node resection might led to false negative results of lymph node metastasis, and therefore, errors in disease staging. Interestingly, more extensive lymphadenectomy was not necessarily accompanied with a better survival outcome in pT1 ESCC.

**Table 2:** Multivariate analysis of T1N0 and T2N0 ESCC patients by Cox proportional regression model.

	T1N0		T2N0	
	HR (95% CI)	p value	HR (95% CI)	p value
**Age, years**		0.143	/	
≤60	Ref.			
>60	1.915 (0.802–4.572)			
**BMI (kg/m^2^)**		0.325		0.8
<18.5	Ref.		Ref.	
18.5–23.9	0.420 (0.120–1.465)		0.973 (0.216–4.376)	
>23.9	0.359 (0.083–1.547)		1.217 (0.656–2.260)	
**Tumor Grade**	/			0.098
Well-differentiated			Ref.	
Moderately differentiated			1.680 (0.703–4.015)	
Poorly or not differentiated			2.805 (1.050–7.495)	
**Lymph node examined (T1/T2)**		0.074		0.077
≤10/≤17	Ref.		Ref.	
11–18/18–24	0.249 (0.074–0.838)		1.157 (0.638–2.099)	
>18/>24	0.621 (0.224–1.720)		0.449 (0.196–0.921)	

### Stepwise Analysis of Added Value Per Resected Lymph Node in the Discovery of LNM

**Figure 5:**
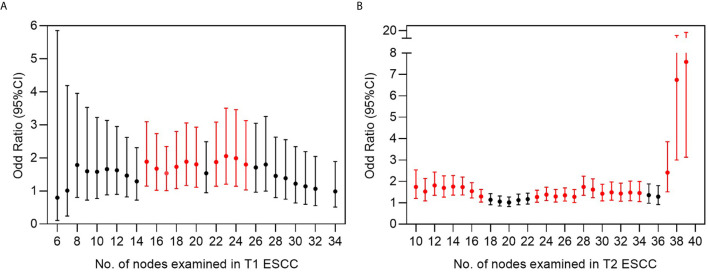
Risk analysis for finding a positive lymph node based on multiple cut-off points of examined lymph nodes in **(A)** T1 and **(B)** T2 ESCC. Odds ratio represents the risk of positive finding on the right side panel of cut-off point compared to the left side panel of cut-off point. Bars in red indicate statistically significant differences by chi-square testing.

All pT1-2NanyM0 patients were included in the stepwise analysis based on a series of cut-off points that divided all patients into two groups: patients with a lower NLNE and those with a higher NLNE (**Figure 5**). Statistically significant odd ratios >1 (colored in red) indicated a higher chance of positive findings of LNM in the group with a higher NLNE. In pT1 ESCC, the added value of a higher NLNE was observed starting from 15 to 25 resected lymph nodes. When the number of lymph nodes resected exceed 25, there was no additional gain in the chance of positive findings (**Figure 5A**). By contrast, patients with pT2 ESCC showed a continuous increase with respect to a chance of positive findings of LNM as the NLNE increased. The aberrant broadening of the confidence interval starting from 37 resected lymph nodes was because of the small samples on the right-side panel (**Figure 5B**).

## Discussion

The question addressed by the present study was whether more extensive resection of regional lymph nodes would really contribute to the survival outcome in patients with relatively early stage ESCC. The potential survival benefit from more extensive lymphadenectomy should be carefully balanced against a decreased postoperative morbidity with less extensive lymphadenectomy. In the current study, data of radical esophagectomy from two thoracic surgical institutions were included to determine the significance of the number of NLNs removed for survival prognosis. Meanwhile, considering that the NLN count information is only available after the surgery, its significance is only to provide qualitative indicators for surgeons and pathologists to set benchmarks and monitor the frequency of outliers. It is challenging to perform the so-called optimal range resection according to the terminal point of NLNs. Therefore, to avoid missing out positive lymph nodes and to obtain a more accurate pathological staging with possibly improved survival outcome after administration of adjuvant therapy, an optimal range of lymph node resection should be explored to provide a reference for surgeons during the operation.

In the current study, none of the node-negative patients enrolled in the survival analysis received neoadjuvant or adjuvant therapies; this eliminated the influence of these therapies on the pathological staging and survival outcome, thereby distinguishing the effects of lymphadenectomy. Our results suggested that the resected number of NLNs do have its prognostic significance in patients with early-stage ESCC. In patients with stage pT1N0 ESCC, the best survival outcome was obtained when the total number of NLNs resection ranged from 10 to 18 (**Figure 4A**). A more extensive resection such as >24 nodes was required to obtain improved OS in pT2N0 ESCC in our study. Interestingly, a similar large-scale study that involved treatment-naïve T1N0M0 and T2-3N0M0 ESCA yielded an inverse result. They suggested that >18 LNs were necessary to maximize survival of T1 disease, while 11–17 LNs provided the same survival advantage as >18 LNs in T2 and T3 disease (22). Indeed, this conclusion was not very convincing given the discrepancy between the results and clinical common sense. Otherwise, the discrepancy between our two studies can result from different methodology and the constitute of histological subtypes. A study enrolling node-negative ESCA patients (including 585 ESCC cases) from China suggested that at least 18 LNs should be resected for accurate staging, given the superior survival outcome in this group of patients. The majority of the participants (87.8%) had stage IIA diseases (T2-3N0M0), showing the same tendency as seen in our results. However, they did not perform subgroup analysis on pathological T1 patients owing to their small sample size (n=56), which probably limited the reliability of their conclusions in this specific group of patients (23).

Based on our further analysis of pT1Nany stage in patients with ESCC, we recommend that the surgeon should remove 15–25 lymph nodes when positive LNM is suspected during operation, to avoid false negative results of LNM. When a stage pT2Nany ESCC is suspected, excision of 24–37 lymph nodes could improve the chance of positive findings of LNM. Evidence for resection of >37 NLNE in pT2 ESCC was not sufficient, given the limited sample size. Since the number of lymph nodes fluctuates over a very wide range in different patients, a recommended range is usually more useful and less restrictive than a definite cut-off point. For practical reasons, we would suggest thoracic surgeons to tailor the lymphadenectomy to the recommended “range” of dissection based on individual characteristics and intraoperative findings. Given the retrospective nature of our study, our primary aim was to provide a quality indicator for both surgeons and pathologists, which might be correlated with more accurate nodal staging and better survival outcome in the aggregate, but not individually. This is the same principle followed by several other previous studies (8, 18, 22). At present, it is still unclear why the number of NLNs can be used as a prognostic factor to predict the potential survival outcome of cancer patients. However, previous studies have suggested that the underlying mechanism may be explained by stage migration or host immune response to cancer cells, as well as the molecular biology of cancer cells (24–26).

The metastatic status of lymph nodes has been recognized as one of the strongest prognostic factors for malignant tumors. ESCA spreads easily through extensive submucosal lymphatic vessels, which means that more extensive lymphadenectomy should improve survival. In Asia, the incidence of ESCC accounts for more than 95% ESCA, and the type of lymph node resection has been standardized, using two- or three-field lymphadenectomy according to the location of the tumor. As per practice guidelines, >15 lymph nodes should be removed during the first esophagectomy (27). A study of 4627 patients with ESCA showed that more extensive lymphadenectomy was accompanied with better survival (28). Additionally, three-field lymphadenectomy showed better 5-year survival rates than two-field lymphadenectomy in the meta-analysis (29, 30). Several other studies also suggested that the number of resected NLNs is an important independent prognostic factor in patients with thoracic ESCC, with increased number of resected NLNs favoring better OS (12, 13, 31).

The extent of lymphadenectomy is still controversial, and the optimal range remains to be determined. Recently, Lagergren et al. demonstrated that ESCA patients with a more extensive lymphadenectomy (21–52 nodes) did not demonstrate a statistically significant reduction in all-cause 5-year mortality than those with only limited lymphadenectomy (0–10 nodes). They concluded that the extent of lymphadenectomy may not affect the 5-year all-cause or disease-specific survival. These results cause the current clinical guidelines to be debatable (20). Similarly, Schaaf et al. found that more extensive lymph node clearance during surgery may not improve survival in 1044 esophageal cancer patients undergoing esophagectomy in Sweden (18). The methodology of these two studies was similar and worth discussing. They treated all the patients with different stages of esophageal cancers in one group and did not stratify to yield specific suggestions. In addition, they categorized the number of lymph node dissection simply using the method of quartering, the design of which was not as good as our study. These might be the primary reasons accounting for the discrepancy of results between studies. In the case of breast cancer, the previously advocated more extensive lymphadenectomy did not actually improve survival, but increased the postoperative morbidities (32). In pancreatic, gastric, or rectal cancer surgery, extensive lymph node dissection has no significant survival benefits (33–37). The potential gain in survival benefit of extended lymphadenectomy may be counteracted by its increased intra- and post-operative complications (33, 35).

Discrepancy about the minimal number of resected lymph nodes exists between different staging systems. An attempt was made to determine the proper range of lymphadenectomy to optimize survival and the accuracy of tumor staging, but no consensus was achieved (38). More importantly, different emphasis on the metastatic nodal station or metastatic nodal number between guidelines from the Japanese Esophageal Society (JES) and AJCC might further create discrepancy. In the current study, the number of resected lymph node stations was positively correlated with the number of resected lymph nodes, and its survival impact might probably echo that of the latter. Therefore, we believe that the number of lymph nodes dissected can be used as a surrogate for the number of stations in the survival analysis. Peyre et al. proposed that at least 23 lymph nodes need to be resected (39). In addition, at least 10 lymph nodes should be removed per the AJCC (40), and 20 lymph nodes per the German S3 guidelines (41). However, some authors believe that the extent of lymphadenectomy should be related to the staging of the tumor. Rizk et al. suggested that at least 10, 20, and 30 lymph nodes should be removed for pT1, pT2, and pT3 diseases, respectively (28). Other authors have suggested that, especially for N0 stage disease, the greater the number of lymph nodes removed, the higher the survival rate, and the higher the lymph node ratio, the higher the survival rate (12, 13, 22, 42).

Our study has some limitations. First, in view of its retrospective nature, the counting and pathological examination of the resected lymph nodes was not fully standardized between two institutions, which might have led to an inaccurate recording of the NLNE. Second, at present, China still lacks a detailed national database of ESCC for external verification, including tumor response and surgical complications. Third, an analysis to explore the optimal number of lymph node dissection that balances the oncological adequacy against operative morbidity was not performed in the current study because of the incompleteness of data on postoperative complications. In future studies, further joint multicenter analyses should be performed and prospective clinical verification of the exact value of and a more appropriate cut-off number of lymph nodes should be carried out.

In conclusion, the optimal extent of lymphadenectomy can be determined by taking both survival outcomes and avoidance of false negative results into consideration. For ESCC patients undergoing radical esophagectomy, the optimal range of NLNE is 15–25 for pT1Nany diseases and 24–37 for pT2Nany diseases.

## Data Availability Statement

The raw anonymous data supporting the conclusions of this article are available from the corresponding authors.

## Ethics Statement

The studies involving human participants were reviewed and approved by Research Ethics Committee Guangdong Provincial people’s Hospital, Guangdong Academy of Medical Sciences (No. GDREC2019687H) and Clinical Research Ethics Committee of the First Affiliated Hospital of Shantou University Medical College(No.2020-094). Written informed consent for participation was not required for this study in accordance with the national legislation and the institutional requirements.

## Author Contributions

Study concepts: HW, WZ, GQ; Study design: WZ, HW; Data acquisition: SH, HW, XG, YZ, ZX, HZ, LX; Quality control of data and algorithms: GQ, RC, ZX, GC; Statistical analysis and interpretation: HW, WZ, RC, XG; Manuscript preparation: HW, WZ, RC, GQ, ZL; Manuscript editing: WZ, HW; Manuscript review: XB, ZZ, RC, GQ, ZL.

## Conflict of Interest

The authors declare that the research was conducted in the absence of any commercial or financial relationships that could be construed as a potential conflict of interest.
